# m^6^A modifications of circular RNAs in ischemia-induced retinal neovascularization

**DOI:** 10.7150/ijms.79409

**Published:** 2023-01-22

**Authors:** Yedi Zhou, Bingyan Li, Zicong Wang, Wei Tan, Jingling Zou, Haixiang Zhou, Yuting Cai, Jie Liu, Yan He, Shigeo Yoshida, Yun Li

**Affiliations:** 1 Department of Ophthalmology, The Second Xiangya Hospital of Central South University, Changsha, Hunan 410011, China.; 2 Hunan Clinical Research Center of Ophthalmic Disease, The Second Xiangya Hospital of Central South University, Changsha, Hunan 410011, China.; 3 National Clinical Research Center for Metabolic Diseases, The Second Xiangya Hospital of Central South University, Changsha, Hunan 410011, China.; 4 Department of Ophthalmology, Kurume University School of Medicine, Kurume, Fukuoka 830-0011, Japan.

**Keywords:** circRNA, m^6^A, oxygen-induced retinopathy, retinal neovascularization, RNA methylation

## Abstract

Ischemia-induced pathological neovascularization in the retina is a leading cause of blindness in various age groups. The purpose of the current study was to identify the involvement of circular RNAs (circRNAs) methylated by N^6^-methyladenosine (m^6^A), and predict their potential roles in oxygen-induced retinopathy (OIR) in mice. Methylation assessment via microarray analysis indicated that 88 circRNAs were differentially modified by m^6^A methylation, including 56 hyper-methylated circRNAs and 32 hypo-methylated circRNAs. Gene ontology enrichment analysis predicted that the enriched host genes of the hyper-methylated circRNAs were involved in cellular process, cellular anatomical entity, and protein binding. Host genes of the hypo-methylated circRNAs were enriched in the regulation of cellular biosynthetic process, the nucleus, and binding. According to the Kyoto Encyclopedia of Genes and Genomes analysis, those host genes were involved in the pathways of selenocompound metabolism, salivary secretion, and lysine degradation. MeRIP-qPCR verified significant alterations in m^6^A methylation levels of mmu_circRNA_33363, mmu_circRNA_002816, and mmu_circRNA_009692. In conclusion, the study revealed the m^6^A modification alterations in OIR retinas, and the findings above shed light on the potential roles of m^6^A methylation in circRNA regulatory functions in the pathogenesis of ischemia-induced pathological retinal neovascularization.

## Introduction

As the shared pathogenesis of proliferative diabetic retinopathy (PDR), retinopathy of prematurity (ROP), and other retinal vasculopathies, ischemia-induced pathological retinal neovascularization is a common cause of irreversible blindness globally in various age groups 1-3. Therefore, effective intervention has become crucial to reducing the severe vision loss caused by retinal neovascularization.

As the most prevalent mRNA modification in mammals 4, N6‑methyladenosine (m^6^A) RNA methylation exerts potential epigenetic functions in various physiological and pathological processes 5-7. We recently revealed the profiles of altered m^6^A epitranscriptomes of mRNAs and long non-coding RNAs in the retinas of oxygen-induced retinopathy (OIR) 8, a classical mouse model for retinal neovascular diseases 9. Several studies suggested the regulatory roles of m^6^A modifications in retinal neovascular diseases. For example, it has been reported that YTHDF2 induces mRNA instability of ITGB1 in an m^6^A‑dependent manner, and attenuated the development of diabetic retinopathy 10. Yao et al. 11 indicated that the m^6^A writer enzyme METTL3 is a promising strategic target for the treatment of pathological neovascularization. Therefore, the potential role of m^6^A methylation in the pathogenesis of retinal neovascularization is worth exploring.

Circular RNA (circRNA) is a closed RNA that lacks 5' and 3' ends 12. Recent studies have revealed important physiological and pathological functions of circRNAs, and their potential as novel molecular biomarkers and therapeutic targets 13. In the vitreous humour of PDR, a total of 131 circRNAs were dysregulated compared to the controls 14. Besides, Li et al. 15 revealed altered exosomal circRNAs in the serum of PDR. In OIR mouse model, 539 circRNAs were significantly changed in the retinal neovascularization group, indicating the possible involvement of circRNA-associated ceRNA networks 16. Significant alteration of circRNAs in peripheral blood mononuclear cells in premature infants suggests they may also be biomarkers and/or therapeutic targets for ROP 17. Numerous studies have revealed profile alterations in m^6^A-modified circRNAs in many diseases, including cerebral infarction 18, severe acute pancreatitis 19, acute myeloid leukemia 20, and oral squamous cell carcinoma 21. Interestingly, Huang et al. 22 recently demonstrated that circRNA circFAT1 enhanced autophagy and reduced pyroptosis in glucose-stressed retinal pigment epithelial cells, and reported the binding of circFAT1 and m^6^A reader YTHDF2.

To date, m^6^A modification of circRNAs in retinal neovascularization remains unknown, and requires further investigation. In the current study, the epitranscriptomic profile of m^6^A-modified circRNAs was established via microarray analysis in the OIR model in mice, followed by validation with MeRIP-qPCR. The potential functions of those circRNAs with altered m^6^A methylation levels were predicted by further bioinformatics analyses.

## Materials & Methods

### Establishment of OIR mouse model

C57BL/6J mice obtained from the SJA Laboratory Animal Center (Changsha, Hunan, China) were used in the study. The OIR model was established as previously described 8, 9. Pups were exposed to hyperoxic conditions (75%) on postnatal day (P) 7, and moved back to room air at P12. Pups in the control group were exposed to room air continuously. At P17, the retinas of mice in both groups were harvested for use in subsequent experiments. Four retinas from two mice were mixed to constitute one sample, and three samples were assessed for microarray analysis of each group. The animal experiments were in accordance with the ARVO Statement and the procedures have been approved by the Institutional Animal Care and Use Committee of the Second Xiangya Hospital of Central South University.

### RNA extraction and m^6^A immunoprecipitation

Total RNA was extracted from retinal tissues using TRIzol (Invitrogen, Carlsbad, CA, USA), the RNA purity and concentration were determined by a NanoDrop ND-1000 spectrophotometer. Either a Bioanalyzer 2100 or Mops electrophoresis was used to assess RNA integrity.

Total RNA and m^6^A spike-in control mixture was mixed with immunoprecipitation (IP) buffer, added the anti-m^6^A rabbit polyclonal antibody (Synaptic Systems, Goettingen, Germany), and then the preparation was incubated at 4°C for 2 h. For each sample, 20 μL Dynabeads M-280 sheep anti-rabbit IgG suspension (Invitrogen) was used, and the preparation was blocked with 0.5% BSA (4°C, 2 h). After washing, they were resuspended in the prepared mixture containing total RNA and antibody and incubated at 4°C for 2 h. After washing, the enriched RNA was eluted with elution buffer at 50°C for 1 h, and then acid phenol-chloroform and ethanol precipitation were used for RNA extraction.

### Epitranscriptomic microarray analysis of circRNAs

IP and Sup RNAs were digested with RNase R (Epicentre, Inc.) to remove linear RNAs and enrich circRNAs. The Super RNA Labeling Kit (Arraystar, Rockville, MD, USA) was used to label Sup RNAs with Cy3, and IP RNAs with Cy5, and then preparations were purified via the RNeasy Mini Kit. The labeled cRNA mixture was fragmented, hybridized on an m^6^A‑circRNA Epitranscriptomic Microarray slide (Arraystar) following the manufacturer's instructions, and scanned by a microarray scanner G2505C (Agilent Technologies, Santa Clara, CA, USA).

Agilent Feature Extraction software (V11.0.1.1) was used to analyze images of captured array. Average of the log2-scaled spike-in RNA intensities were used for normalization of the IP and Sup raw intensities. m^6^A quantities were calculated based on normalized IP amounts, and m^6^A methylation levels were calculated as the percentage of modification in the input according to normalized intensities. Raw data from the microarray analysis were deposited in the database of Gene Expression Omnibus (No. GSE213362). The associations between circRNA m^6^A methylation and expression levels were conducted by the intersection of the current m^6^A microarray data and the expression profile of circRNAs in OIR retinas in accordance with a previous study by our research group 16.

### Methylated RNA immunoprecipitation-qPCR

Methylated RNA immunoprecipitation (MeRIP)-qPCR analysis was conducted to validate the microarray data. SuperScript^TM^ III Reverse Transcriptase (Invitrogen) was used to synthesize the first-strand cDNA from IP RNA, and the system of QuantStudio^TM^ 5 Real‑Time PCR (Applied Biosystems, Foster City, CA, USA) with 2X PCR master mix (Arraystar) was used to conduct RT-qPCR. The IP fraction in the input was calculated as MERIP/input (%) as described in Xing et al. 23. Data are presented as means ± SEM. Primer sequences are displayed in Table 1.

### Bioinformatics analyses

Analyses of gene ontology (GO) and Kyoto Encyclopedia of Genes and Genomes (KEGG) were used to reveal the functional annotation and the pathways involved. The network of circRNA-miRNA-mRNA was constructed under the competing endogenous RNA (ceRNA) hypothesis.

### Statistical analysis

The differences between the two groups were compared by Student's t-test. Significant differences in m^6^A-methylated circRNAs between the two groups in microarray analyses were determined by fold change ≥ 1.5 and *p* < 0.05. For MeRIP-qPCR data, *p* < 0.05 was deemed to indicate statistical significance.

## Results

### OIR alters the profile of m^6^A methylation levels in circRNAs

**Figure 1:**
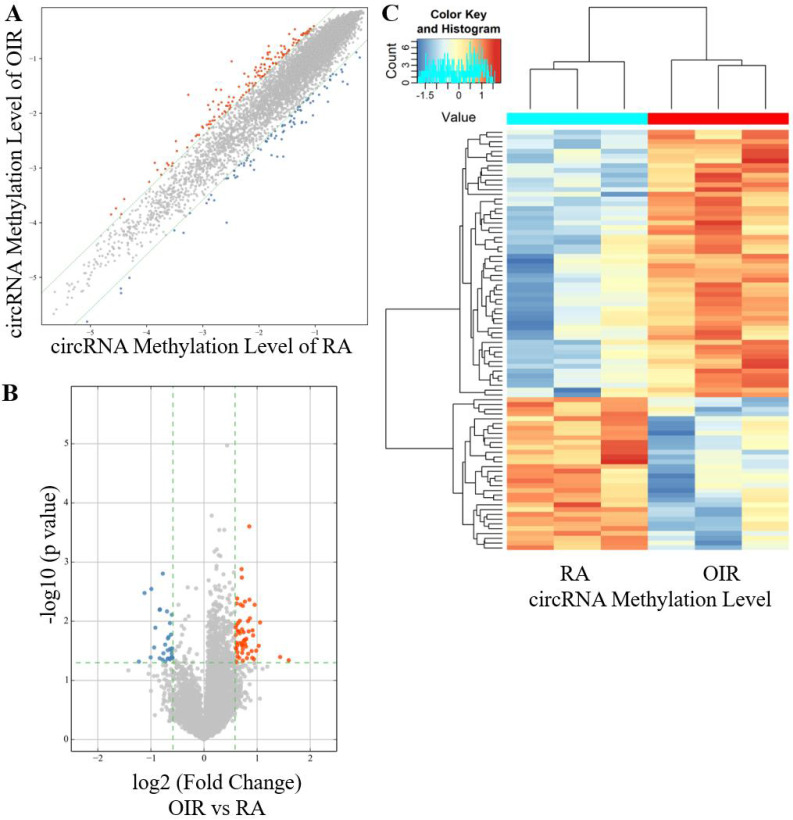
** CircRNA m^6^A methylation levels in retinas of OIR and room air control groups. (A)** Scatter plot showing correlation distribution of circRNA m^6^A methylation level in OIR mice and room air (RA) controls. The red and blue dots represent the hyper- and hypo-methylated circRNAs. The gray lines indicate the default alteration of 1.5-fold. **(B)** Volcano plot showing significantly hyper-methylated and hypo-methylated circRNAs in OIR retinas compared to RA controls. The red and blue dots represent the hyper- and hypo-methylated circRNAs with statistical significance (fold change ≥ 1.5 and *p* < 0.05). **(C)** Heatmap of hierarchical clustering showing differentially methylated circRNAs in OIR retinas compared to RA controls (*n* = 3 per group).

A total of 88 circRNAs were differentially modified by m^6^A methylation in the retinas of the OIR group and the control group, including 56 hyper-methylated circRNAs and 32 hypo-methylated circRNAs in the OIR group (Figure 1A-1C). The top ten hyper-methylated and hypo-methylated circRNAs are listed in Table 2.

### Associations between circRNA m^6^A methylation and expression levels

**Figure 2:**
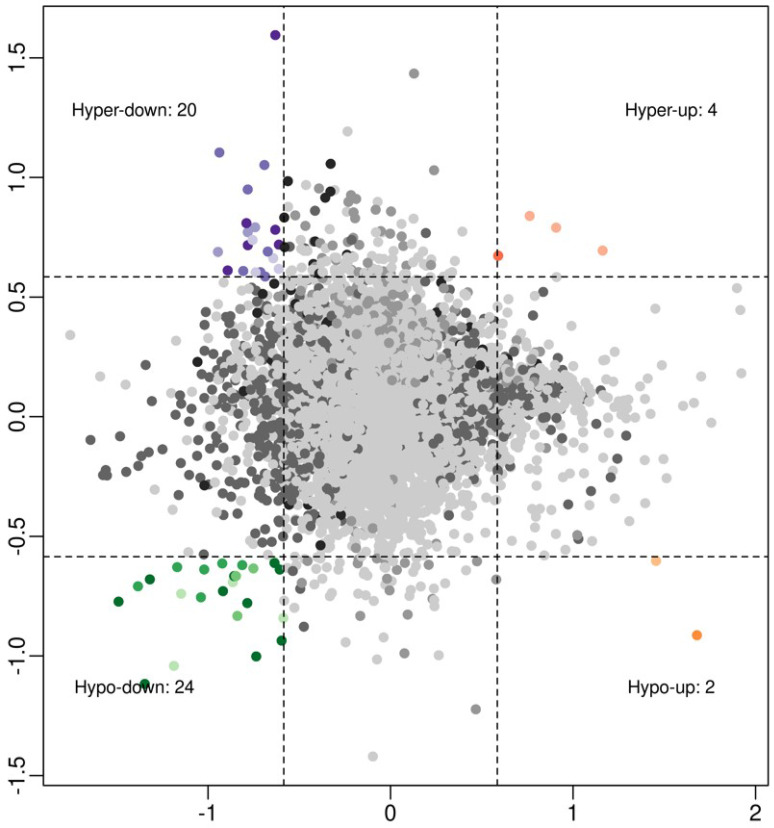
Association analysis of circRNA m^6^A methylation levels and expressions in OIR retinas: 4 hyper-up circRNAs, 20 hyper-down circRNAs, 2 hypo-up circRNAs, and 24 hypo-down circRNAs (fold change ≥ 1.5).

To investigate associations between circRNA expression and m^6^A methylation levels, circRNAs were intersected with altered m^6^A methylation and differentially expressed circRNAs by a previous study 16. Using a threshold of ≥ 1.5-fold change, four interaction modes were identified (Figure 2), including 4 hyper-methylated and upregulated circRNAs, 20 hyper‑methylated and downregulated circRNAs (6 circRNAs met the significance threshold of *p* < 0.05 in both methylation and expression), 2 hypo-methylated and upregulated circRNAs, and 24 hypo‑methylated and downregulated circRNAs (10 circRNAs met the significance threshold of *p* < 0.05 in both methylation and expression).

### Predictions of involved functions and pathways in host genes of the altered m^6^A-modified circRNAs

**Figure 3:**
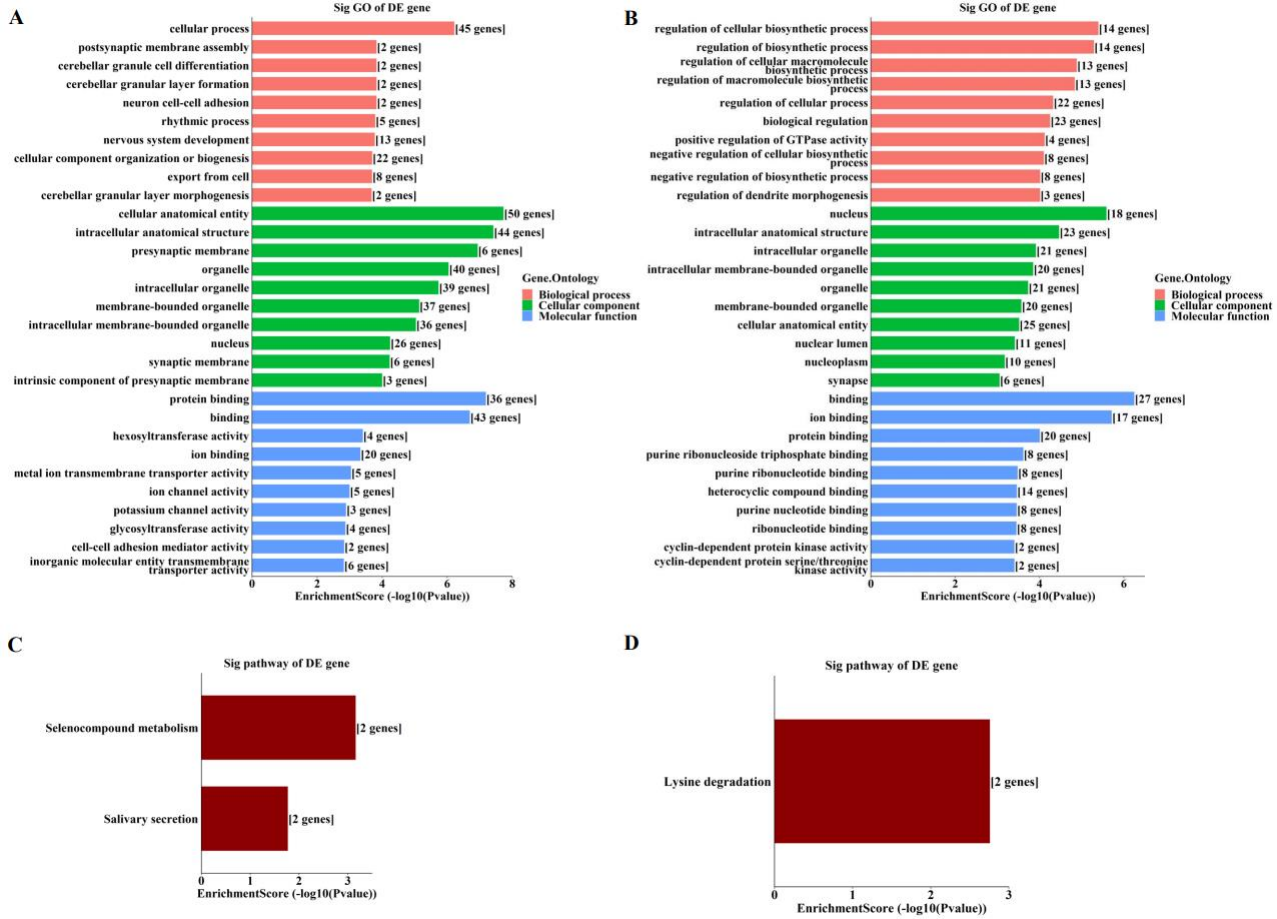
** Enrichment analyses of host genes of significantly altered m^6^A-modified circRNAs in OIR retinas. (A, B)** GO analysis of host genes of significantly hyper-methylated (A) and hypo-methylated (B) circRNAs. **(C, D)** KEGG analysis of host genes of significantly hyper-methylated (C) and hypo-methylated (D) circRNAs. The bar plots display the top enrichment scores of the significant enrichment terms.

By GO enrichment analyses, host genes of the enriched hyper‑methylated circRNAs were involved in cellular process, cellular anatomical entity, and protein binding (Figure 3A). Those of the enriched hypo-methylated circRNAs were involved in the regulation of cellular biosynthetic process, the nucleus, and binding (Figure 3B). KEGG analyses identified only three pathways, with host genes of hyper-methylated circRNAs involved in selenocompound metabolism and salivary secretion (Figure 3C), and those of hypo-methylated circRNAs involved in lysine degradation (Figure 3D).

### Verification of altered methylation levels by MeRIP-qPCR

**Figure 4:**
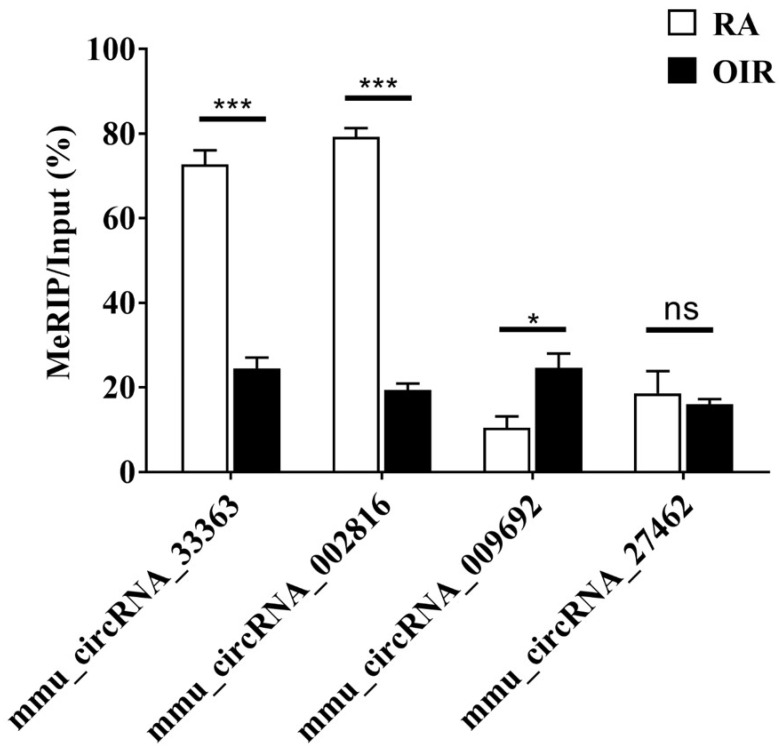
M^6^A methylation levels of mmu_circRNA_33363, mmu_circRNA_002816, mmu_circRNA_009692, and mmu_circRNA_27462 determined by MeRIP-qPCR. The data were presented as mean ± SEM (*n* = 3 per group). *p < 0.05; ***p < 0.001; ns, not significant.

m^6^A methylation levels of four selected circRNAs assessed via MeRIP-qPCR are shown in Figure 4. The m^6^A methylation levels of mmu_circRNA_33363 and mmu_circRNA_002816 significantly decreased in OIR group, and m^6^A methylation level of mmu_circRNA_009692 was significantly increased (*p* < 0.05). The m^6^A methylation level of mmu_circRNA_27462 was slightly downregulated, but of no statistical significance (*p* > 0.05). The downward trend was consistent with the microarray analysis.

### ceRNA analysis and further predictions

**Figure 5:**
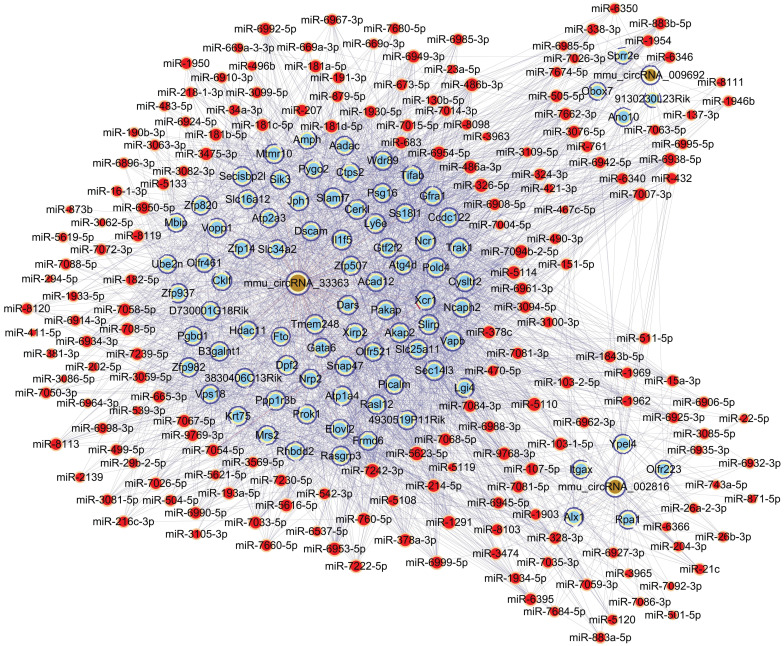
Construction of the circRNA-miRNA-mRNA network under ceRNA hypothesis based on three validated circRNAs with significantly altered m^6^A methylation levels. Light blue nodes represent mRNAs, red nodes represent miRNAs, and brown nodes represent circRNAs with significantly altered m^6^A methylation levels. Edges with T-shaped arrows and those without arrows indicate directed and undirected relationships, respectively.

**Figure 6:**
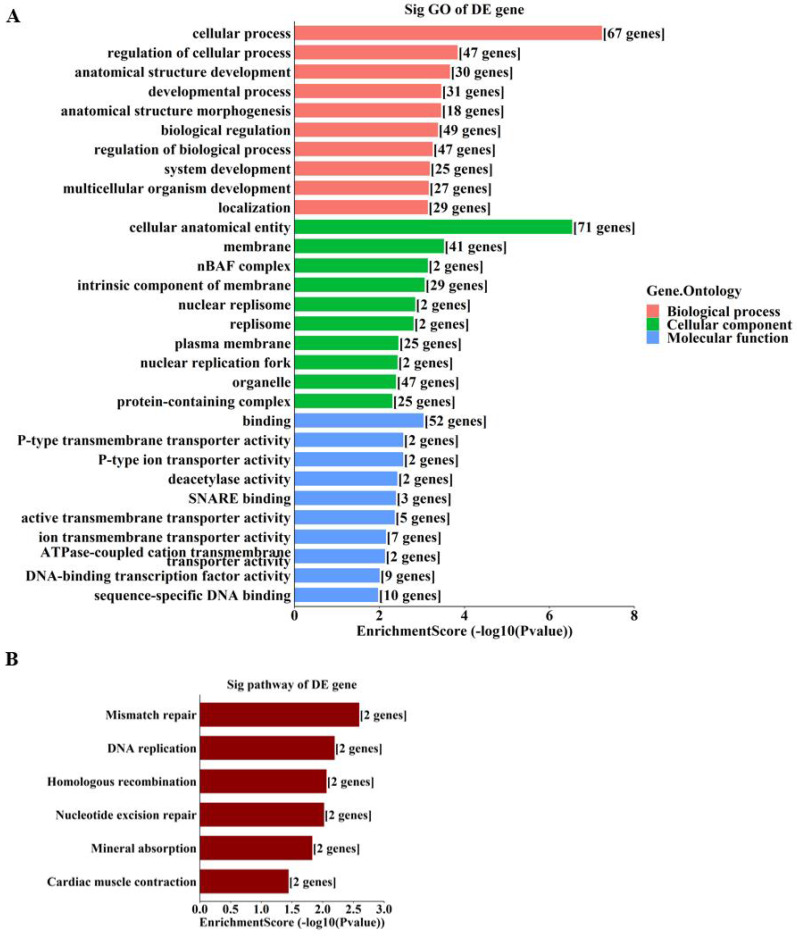
** Enrichment analyses of involved genes from the ceRNA network. A.** GO analysis of involved genes from the ceRNA network. **B.** KEGG analysis of involved genes from the ceRNA network. The bar plots display the top enrichment scores of the significant enrichment terms.

To further unravel the mechanisms of the above-described circRNAs, a circRNA-miRNA-mRNA network was constructed under ceRNA hypothesis. The network was composed of 272 nodes (3 circRNAs, 185 miRNAs, and 84 mRNAs) and 2697 edges (2613 directed edges and 84 undirected edges) (Figure 5). Based on the involved mRNAs, GO analysis identified the top enriched terms, including “cellular process”, “cellular anatomical entity”, and “binding” (Figure 6A). KEGG pathway analysis identified the top pathways involved, including mismatch repair, DNA replication, and homologous recombination (Figure 6B).

## Discussion

M^6^A modification, as an essential epigenetic regulator, has been suggested to involve in vision function and pathogenesis of ophthalmic diseases 24. Recently, with the wide application of high-throughput sequencing technologies, there is accumulating evidence that crosstalk between m^6^A modifications and circRNAs contributes to multifaceted physiological and pathological processes 25, 26. Therefore, a better understanding of m^6^A-modified circRNAs in the pathogenesis of retinal neovascularization is warranted.

The current study revealed the epitranscriptomic profile of m^6^A-modified circRNAs in OIR retinas. A total of 88 circRNAs with significantly altered m^6^A modifications were identified (Figure 1). The study also identified enriched biological functions via GO analysis, and the pathways involved via KEGG analysis, based on the host genes of hyper-methylated and hypo-methylated circRNAs (Figure 3). These data and predictions shed light on the potential functions of m^6^A modifications in circRNA regulation of retinal neovascularization. For example, “lysine degradation” was an enriched pathway of the host genes of hypo-methylated circRNAs. Dong et al. 27 demonstrated the presence and involvement of acrolein-lysine adduct in fibrovascular membrane in PDR patients. In our previous study, lysine was significantly upregulated in retinal tissues of mice with OIR 28. Lysine degradation occurs mainly via saccharopine formation and the pipecolic acid pathway, and involves interaction with subcellular compartmentalization and enzyme deficiencies 29. However, the specific roles of lysine degradation and the mechanisms involved require further investigation.

It has been suggested that understanding ceRNA crosstalk provides extraordinarily essential views of the regulatory roles of circRNAs 30. A recent study indicated that m^6^A modification promotes the capability of binding of circALG1—an overexpressed circRNA in colorectal cancer—to miR-342-5p, enhancing ceRNA regulation, which may facilitate the development of a novel therapeutic method targeting colorectal cancer 31. Li et al. 32 reported that m^6^A‑dependent upregulation of circMETTL3 aggravated breast cancer progression by acting as a ceRNA via the circMETTL3/miR-31-5p/CDK1 axis. In the current study, MeRIP-qPCR results indicated significant alteration of m^6^A methylation levels of mmu_circRNA_33363, mmu_circRNA_002816, and mmu_circRNA_009692 (Figure 4). Therefore, a circRNA-miRNA-mRNA network was constructed under hypothesis of ceRNA based on these three validated circRNAs (Figure 5). GO analysis based on the 84 mRNAs involved showed that the majority of the enriched genes were involved in cellular process, cellular anatomical entity, and binding (Figure 6A); indicating the potential importance of m^6^A-modified circRNAs in retinal neovascularization. Recent studies showed the involvement of RNA editing modifications in retinal and vascular pathogenesis 33, 34, which indicated that the differential edited genes were enriched in pathways associated with angiogenesis, inflammation and apoptosis. It is also interesting to conduct the integrated analysis of multi-omics of the m^6^A methylation with other kinds of RNA modifications to further explore the cellular mechanisms in ischemia-induced retinopathy.

In summary, the present study investigated the m^6^A modification alterations of circRNAs in the retinas of OIR. The possible relevant functions and pathways were also predicted by bioinformatics analyses. These provide novel insight into the involvement of m^6^A methylation in circRNA regulatory functions in OIR. Further investigations are needed to reveal the m^6^A epitranscriptomic profiles of circRNAs in clinical samples from retinal neovascular diseases, and to identify the specific roles of these m^6^A-modified circRNAs in the pathological process of ischemia-induced retinal neovascularization.

## Figures and Tables

**Table 1:** Primer sequences of the circRNAs validated by MeRIP-qPCR

CircRNA	Forward/reverse primers	Tm (°C)	Product length (bp)
mmu_circRNA_33363	F: 5' GTGATCTCAGTATTCAAAGGTGT 3' R: 5' CTCATGGTTTCCTATGGTATT 3'	60	191
mmu_circRNA_002816	F: 5' AGAAGCCCATAAGTAAACAAGG 3' R: 5' AAAATTCTCCACTTTATCCGTT 3'	60	88
mmu_circRNA_009692	F: 5' CAAACCTCACAAAGCACAATATC 3' R: 5' CGAAGAACCTGAACCTGCTG 3'	60	73
mmu_circRNA_27462	F: 5' ATCCGAAGACTCTGTGAAACTGT 3' R: 5' CTTGTTTACTTATGGGCTTCTTCA 3'	60	126

**Table 2:** Top 10 hyper-methylated and hypo-methylated circRNAs in OIR retinas

CircRNA	Regulation	Fold change	*p* value	Chrom	CircRNA type
mmu_circRNA_002170	hyper	3.020529	0.045852	15	intergenic
mmu_circRNA_007369	hyper	2.701580	0.040396	15	exonic
mmu_circRNA_42751	hyper	2.079537	0.010493	8	sense overlapping
mmu_circRNA_40551	hyper	2.040813	0.026124	6	exonic
mmu_circRNA_29908	hyper	1.977367	0.031570	16	exonic
mmu_circRNA_42252	hyper	1.935942	0.005281	7	exonic
mmu_circRNA_43770	hyper	1.918786	0.043799	9	intronic
mmu_circRNA_32467	hyper	1.898660	0.017431	19	exonic
mmu_circRNA_010066	hyper	1.884827	0.014267	2	exonic
mmu_circRNA_38600	hyper	1.877949	0.041738	5	exonic
mmu_circRNA_21859	hypo	2.340441	0.048265	10	intronic
mmu_circRNA_33363	hypo	2.174187	0.003338	2	exonic
mmu_circRNA_33864	hypo	2.007061	0.041051	2	exonic
mmu_circRNA_22928	hypo	1.989066	0.002853	11	exonic
mmu_circRNA_26912	hypo	1.916923	0.027862	13	exonic
mmu_circRNA_19661	hypo	1.887089	0.012903	1	exonic
mmu_circRNA_35922	hypo	1.784253	0.042428	3	exonic
mmu_circRNA_19595	hypo	1.784158	0.006434	1	exonic
mmu_circRNA_26987	hypo	1.777264	0.006297	13	exonic
mmu_circRNA_27462	hypo	1.718866	0.044697	14	exonic
